# Stabilization of HIF-1α alleviates osteoarthritis via enhancing mitophagy

**DOI:** 10.1038/s41419-020-2680-0

**Published:** 2020-06-25

**Authors:** Sunli Hu, Chunwu Zhang, Libin Ni, Chongan Huang, Dingwen Chen, Keqing Shi, Haiming Jin, Kairui Zhang, Yao Li, Ling Xie, Mingqiao Fang, Guangheng Xiang, Xiangyang Wang, Jian Xiao

**Affiliations:** 1 Department of Orthopaedics, The Second Affiliated Hospital and Yuying Children’s Hospital of Wenzhou Medical University, Wenzhou, Zhejiang China; 2 Molecular Pharmacology Research Center, School of Pharmaceutical Science, Wenzhou Medical University, Wenzhou, Zhejiang China; 3 The Second School of Medicine, Wenzhou Medical University, Wenzhou, Zhejiang China; 4 Department of Orthopaedics, The first Affiliated Hospital of Wenzhou Medical University, Wenzhou, Zhejiang China

**Keywords:** Mitophagy, Osteoarthritis

## Abstract

Mitochondrial dysfunction leads to osteoarthritis (OA) and disc degeneration. Hypoxia inducible factor-1α (HIF-1α) mediated mitophagy has a protective role in several diseases. However, the underlying mechanism of HIF-1α mediated mitophagy in OA remains largely unknown. This current study was performed to determine the effect of HIF-1α mediated mitophagy on OA. Therefore, X-ray and tissue staining including HE staining, safranin O-fast green (S-O) and Alcian Blue were used to assess imageology and histomorphology differences of mouse knee joint. Transcriptional analysis was used to find the possible targets in osteoarthritis. Western blot analysis, RT-qPCR and immunofluorescence staining were used to detect the changes in gene and protein levels in the vitro experiment. The expression of HIF-1α was increased in human and mouse OA cartilage. HIF-1α knockdown by siRNA further impair the hypoxia-induced mitochondrial dysfunction; In contrast, HIF-1α mediated protective role was reinforced by prolylhydroxylase (PHD) inhibitor dimethyloxalylglycine (DMOG). In addition, HIF-1α stabilization could alleviate apoptosis and senescence via mitophagy in chondrocytes under hypoxia condition, which could also ameliorate surgery-induced cartilage degradation in mice OA model. In conclusion, HIF-1α mediated mitophagy could alleviate OA, which may serve as a promising strategy for OA treatment.

## Introduction

Osteoarthritis (OA) is a leading cause of disabling disease worldwide^1^. It imposes a huge burden on the family affected, social welfare institutions and the social economic cost^2^. The incidence appears to be increased in the past decade. Especially, the incidence of osteoarthritis increased sharply in the elderly people^3^. Clinically, knee joint is the most common site of arthritis, followed by the hand and hip^4^. However, pain is the most common clinical symptom of osteoarthritis^5^. When noninvasive and conservative treatment fails to achieve relieving symptom, surgery is the only last choice for patients^6^.

Mitochondria is one of the most complex and important organelles found in eukaryotic cells and carries out several biochemical processes, maintains energy production through adenosine triphosphate (ATP) generation^7^. Mitochondrial dysfunction is thought to be associated with extracellular matrix metabolism, apoptosis, aging and a range of pathological processes, including arthritis and disc degeneration^8,9^.

Autophagy is an important part of cellular process, which has dual role in chondrocyte fate. Studies have revealed the cytoprotective role of autophagy in OA development, while it also can lead to autophagic cell death^10–12^. Several researchers strengthened knowledge of the protective role of autophagy against both chondrocyte apoptosis and OA development. Autophagy-related proteins such as Beclin-1and LC3, were markedly increased in human chondrocytes and associated with increased apoptosis^10^. In an OA mouse model, Carames et al. showed that OA development is accompanied by a loss of key regulators of autophagy and an increase of apoptosis, as determined by PARP cleavage^11^. Administration of rapamycin alleviated the severity of experimental OA^13^ while silencing of beclin-1 resulted in enhanced chondrocyte death. Mitophagy is a special form of autophagy that maintains mitochondrial homeostasis via eliminating impaired organelles, unwanted protein and reducing cellular stress caused by harmful stimulus^14^. Basal levels of mitophagy maintain cellular homeostasis and protect cells. During several cellular stress including high glucose, oxidative stress and low-oxygen conditions, mitophagy can be stimulated whereby up-regulated mitophagy level can promote cell survival by removing damaged mitochondria^15^. Mitophagy has been associated with mitochondrial dysfunction and apoptosis in the pathological process of several diseases, including heart disease^16^, neurodegenerative diseases^17^ and kidney diseases^18^.

Hypoxia inducible factor-1 (HIF-1) is a heterodimer composed of oxygen-sensitive HIF-1α and constitutively expressed HIF-1β subunits, regulates adaptive responses to hypoxia conditions^19–21^. Under normal conditions, the HIF-1α subunit is rapidly degraded by prolyl-4-hydroxylases (prolyl hydroxylation domain protein, PHD), a major biodegradable small molecule of HIF-1α. PHD is less active during hypoxia or ischemia^22,23^, which leads to inhibiting the HIF-1α degradation and causing the HIF-1α to transfer into the nucleus whereas it recruits HIF-1β, inducing the expression of specific target genes, such as BNIP3, an essential molecule for mitophagy^23,24^. In addition, the damaged mitochondria clearance by mitophagy can prevent from ROS synthesis and apoptotic cell death^25^.

HIF-1α plays a significant role in chondrocyte survival^26^. HIF-1 α was detected in the nuclear extracts of chondrocytes which were isolated from normal or OA cartilages and cultivated under normoxic condition. HIF-1α conditional KO led to massive chondrocyte cell death in the growth plate^27^. Bohensky et al. suggested that prior to their apoptotic cell death, promoting chondrocytes survival in an autophagic state, which was stimulated by HIF-1α^28^. The mechanisms of HIF-1 α induced autophagy contains modulation of beclin-1/Bcl-2 complex^28^ and inhibition of mTOR^29^. Recently, Chen et al. demonstrated the potential role of Bcl-2 modulation in HIF-1α-mediated autophagy^30^. HIF-1α/HIF-2α imbalance is a main regulator of chondrocyte survival/death. HIF-1α expression is decreased and HIF-2α is increased, skewing the imbalance resulting in autophagy and apoptosis. Unlike HIF-1α, HIF-2α is a negative regulator of the autophagy. HIF-2α-silenced chondrocytes exhibited high elevation of lysosomal activity, inhibition of mTOR expression and the presence of autophagosomes. Previous studies failed to elaborate the relationship between HIF-1α and mitophagy in osteoarthritis. Downstream signaling mechanisms of mitophagy in osteoarthritis are remained unclear until now. In our present study, we identified that hypoxia induced mitophagy could protect chondrocyte from apoptosis, and senescence. HIF-1α overexpression could enhance mitophagy in vitro experiments. DMOG mediated HIF-1α up-regulation inhibits the DMM-induced cartilage degradation. In summary, HIF-1α may become a potential target for treating osteoarthritis.

## Results

### HIF-1α was increased in human and mouse knee articular cartilage

**Fig. 1:**
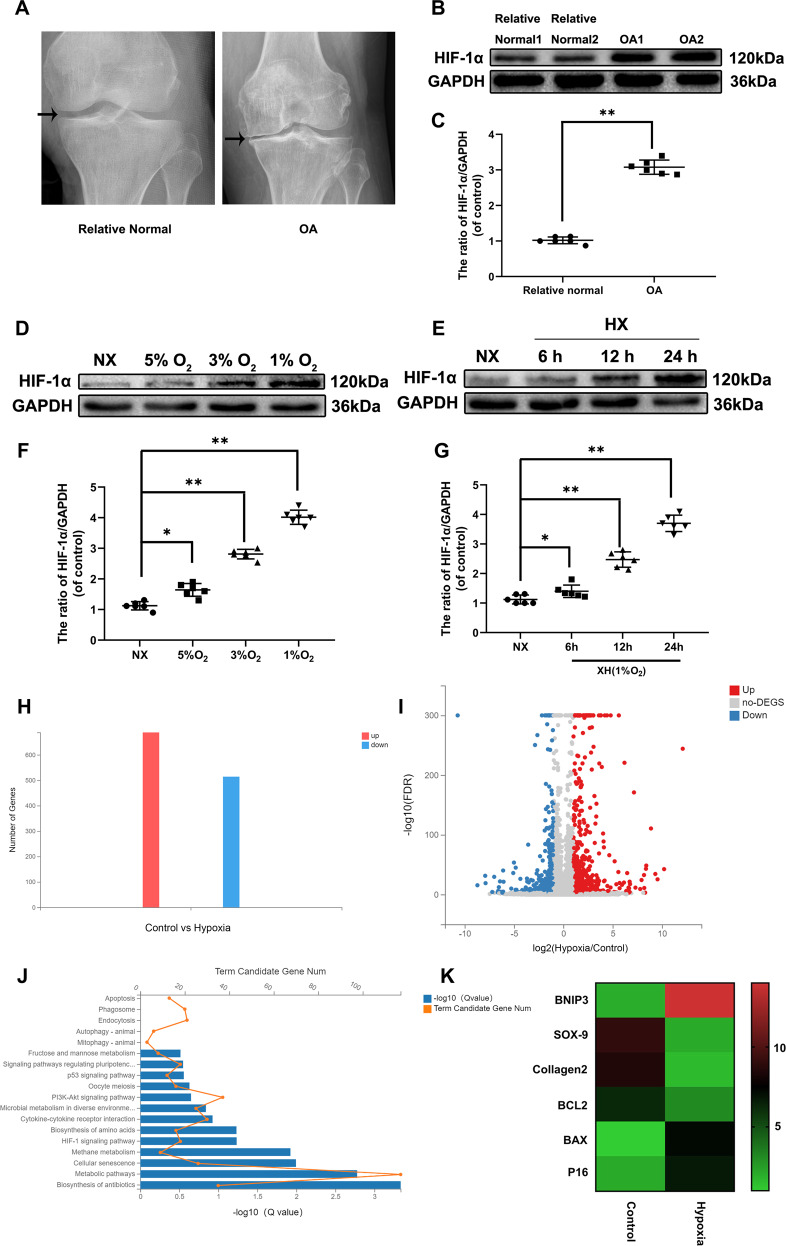
HIF-1α is increased in human and mouse knee articular cartilage. **a** Representative X ray images of normal and osteoarthritis patients. **b** The protein expression of HIF-1α in human chondrocytes derived from normal and osteoarthritis patients. **c** Quantification of HIF-1α by immunoblots. **d**, **e** The expression of HIF-1α from hypoxia treated mouse chondrocytes was analyzed by western blot. **f**, **g** Quantification of HIF-1α immunoblots. **h**–**k** Bioinformatic histogram, Volcano Plot, KEGG and Heatmap analyses of the key molecules and signaling pathways differentially regulated in mouse chondrocytes treated with hypoxia condition (1% pO_2_) for 24 h based on transcriptome analysis. All data represent mean ± S.D. (*n* = 6). **P < 0.01, *P < 0.05.

Previous studies show that mitophagy induces chondrocytes degeneration, which indicated that HIF-1α is related to the OA development. To confirm the relationship between OA and HiF-1α, western-blot analysis was performed in the chondrocytes in human knee cartilage tissue of normal and OA patients. X-ray images of normal and OA patient were displayed in Fig. 1a. Therefore, our significant results showed that HIF-1α expression level was enhanced in human OA group compared to the healthy control (Fig. 1b, c). Besides, to verify the HIF-1α changes in mouse chondrocytes in vitro, the mouse chondrocytes were cultured in incubators with different oxygen concentrations (1, 3, 5% pO_2_) for 24 h and hypoxia condition (1% pO_2_) for 6, 12, 24 h (Fig. 1d–g). Then, the HIF-1α protein expression levels were measured in each group by western-blot analysis. We found that the HIF-1α protein expression levels were considerably elevated in the time-dependent manner and under hypoxia. The HIF-1α level expression levels were enhanced in chondrocytes cultured in hypoxia condition (1% pO_2_) for 24 h. Thus, we selected this condition for the subsequent experiments. To elucidate further biological manners of chondrocytes in hypoxia condition, RNA sequencing was conducted to explore the transcription profiles of mouse chondrocytes treated with hypoxia stimulation for 24 h or not. Total 1202 differently expressed genes were identified (including up and down genes) between Control vs Hypoxia group (Fig. 1h, i). According to the functional analysis of dysregulated mRNAs, we found that metabolic pathways, apoptosis, autophagy and cellular senescence which were tightly associated with osteoarthritis (Fig. 1j). Interestingly, BNIP3 interacts with Beclin inducing mitophagy was markedly up-regulated and enriched (Fig. 1k). Thus, these substantial data suggested that HIF-1α was augmented in OA and hypoxia condition and the increased HIF-1α expression regulated mitophagy, apoptosis and senescence makers.

### Hypoxia stimulated mitochondrial ROS generation, apoptosis, and autophagy in chondrocytes

**Fig. 2:**
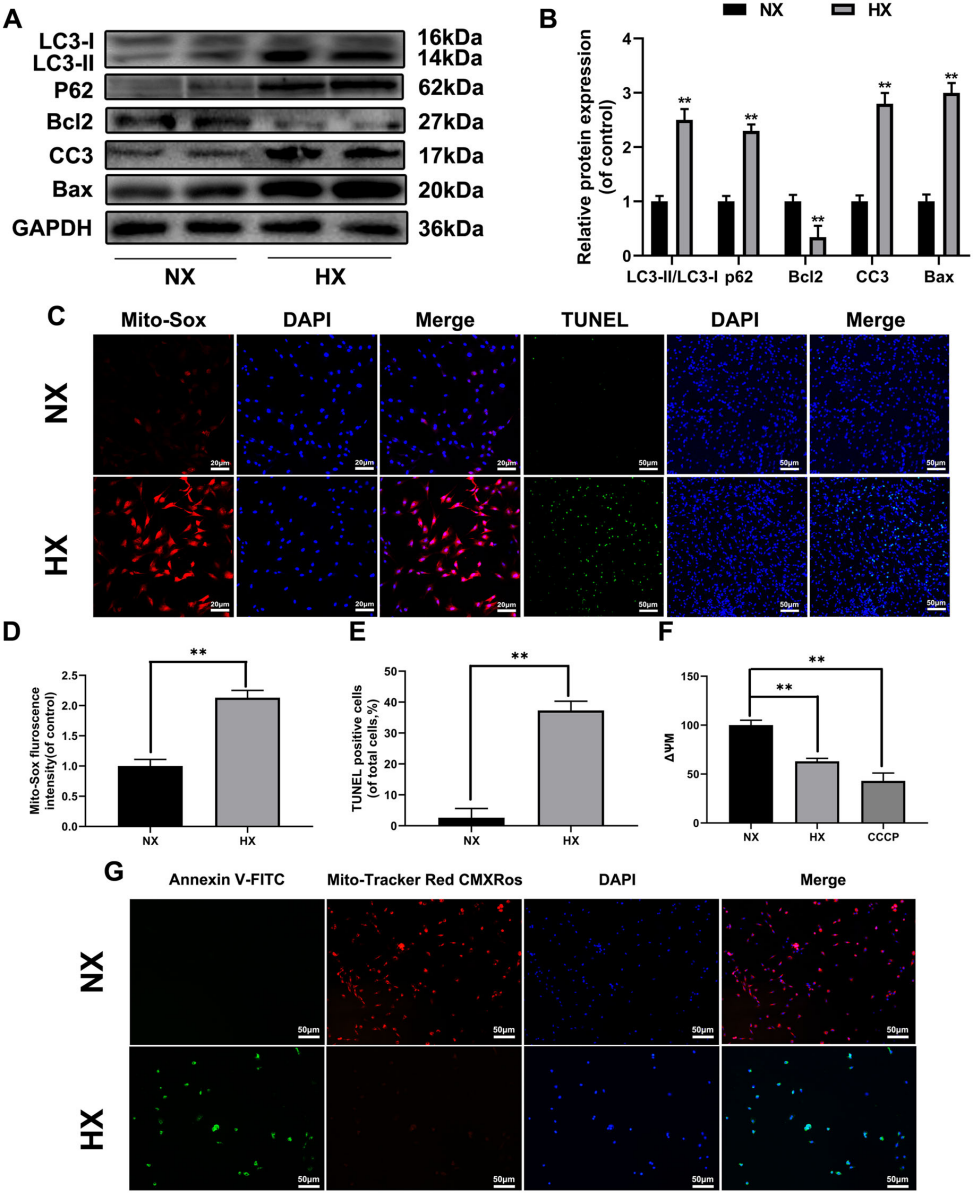
Hypoxia stimulates mitochondrial ROS generation, apoptosis, and autophagy in chondrocytes. Chondrocytes stimulated with hypoxia condition(1% pO_2_) for 24 h. **a** The expression of LC3-I, II, P62, Bcl-2, Bax and Cleaved-caspase3 from hypoxia treated chondrocytes were analyzed by western blot. **b** Quantification of LC3-I, II, P62, Bcl-2, Bax and cleaved-caspase 3 by immunoblots. **c**–**e** Hypoxia induced mitochondria ROS and apoptosis in chondrocytes were analyzed by Mito-Sox stains and TUNEL assay. The fluorescence of Mito-Sox and TUNEL was quantified. **f** ΔѰM loss was measured by JC-1 and analyzed by flow cytometer. CCCP was used as positive control. **g** The Mito-tracker red probe was used to measure the mitochondrial membrane potential (MMP). Annexin V-FITC was used to assess apoptosis. All data represent mean ± S.D. (*n* = 6). **P < 0.01, *P < 0.05.

Fluorescence staining and western-blot were performed to detect mitochondrial ROS generation, apoptosis and autophagy respectively in hypoxia-stimulated chondrocytes. Compared with control group, the Bax, cleaved-caspase3 and Cytochorome C (Cyto C) protein expression levels were significantly increased in hypoxia stimulated group, while Bcl-2 (the anti-apoptotic protein) expression level was significantly decreased (Fig. 2a, b). Moreover, western blotting data showed that hypoxia not only led to apoptosis but also induced autophagy (Fig. 2a, b). The LC3-II/I ratio was significantly increased while p62 was elevated in hypoxia-treated chondrocytes, indicating that autophagy flux was impaired. In order to confirm autophagy activation, bafilomycin is an autophagosome degradation inhibitor used to quantize autophagic flux activity. Our remarkable results demonstrated that autophagy was activated (Fig S1). The mitochondrial ROS level was significantly increased in hypoxia condition (1% pO_2_, 24 h), as well as changes in mitochondrial transmembrane potential, leading to mitochondrial damage (Fig. 2c–e, g). TUNEL assay and Annexin V-FITC revealed that hypoxia could stimulate apoptosis (Fig. 2c, e, g). JC-1 is an ideal fluorescent probe widely used to detect mitochondrial membrane potential (mitochondrial be potential) ΔΨm. The jc-1 transformation from red fluorescence to green was an early apoptosis indicator, suggesting that hypoxia can impair mitochondrial bioenergetics via decreasing ΔѰM (Fig. 2f).

In summary, these substantial findings indicated that hypoxic environment could cause chondrocytes apoptosis, activate autophagy and enhance mitochondrial ROS.

### HIF-1α was associated with hypoxia-induced chondrocytes self-protection

**Fig. 3:**
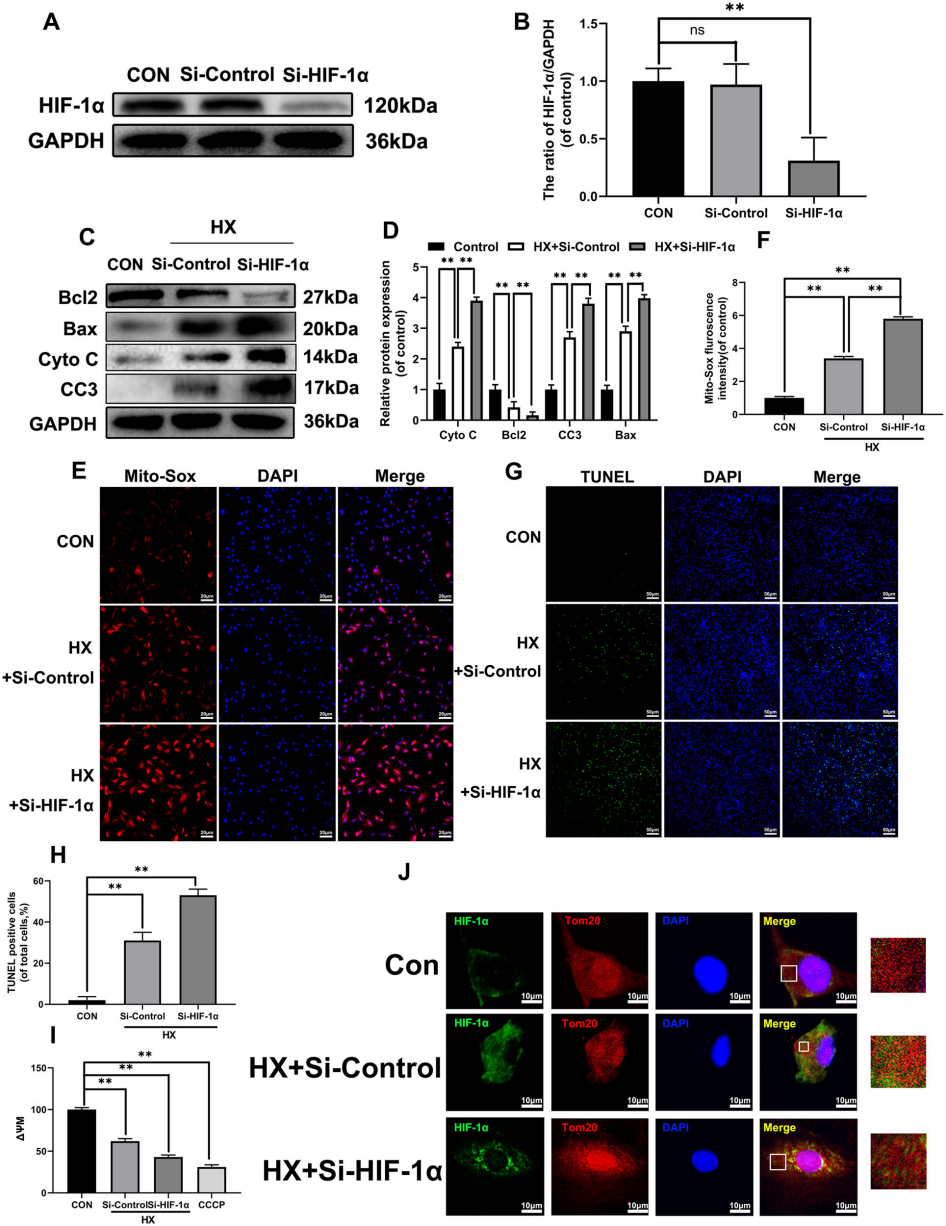
HIF-1α is associated with hypoxia-induced chondrocytes self-protection. The chondrocytes were pretreated with HIF-1α target siRNA for 48 h followed hypoxia treatment (1% pO_2_) for 24 h. **a** The expression of HIF-1α was evaluated by western blots. **b** Quantification of HIF-1α immunoblots. **c** The expression of apoptotic proteins, including Bcl-2, Bax, Cyto C, and cleaved-caspase3 was evaluated by western blots. **d** Quantification of Bcl-2, Bax, Cyto C, and Cleaved-caspase 3 by immunoblots. **e** Mito-Sox was performed in chondrocytes. **f** The fluorescence intensity of Mito-sox in chondrocytes was quantified. **g** TUNEL assay was performed in chondrocytes. **h** The fluorescence intensity of TUNEL in chondrocytes was quantified. **i** JC-1 was measured in chondrocytes. **j** Representative image of immunofluorescence double staining of HIF-1α and Tom20 in chondrocytes. All data represent mean ± S.D. (*n* = 6). **P < 0.01, *P < 0.05.

HIF-1α expression levels were altered in hypoxia-treated chondrocytes. To further verify whether HIF-1α played a protective role in hypoxia-induced OA, mitochondrial function, apoptosis and autophagy related indicators were assessed in rat chondrocytes transfected HIF-1α siRNA (as shown in Fig. 3a, b). HIF-1α expression levels were significantly inhibited. In addition, the Bax, cleaved-caspase3 and Cytochrome C protein expression levels were further increased using HIF-1α knockdown while Bcl2 expression levels were significantly augmented (Fig. 3c, d). The results showed that HIF-1α-knockdown further increased hypoxic-induced apoptosis and mitochondrial ROS production as well as decreased ΔѰM in chondrocytes, demonstrating that decreased HIF-1α expression level impaired energy production and mitochondrial functions (Fig. 3e, f, i). TUNEL also confirmed that HIF-1α knockdown promoted hypoxia-induced apoptosis (Fig. 3g, h). Double staining of HIF-1α and Tom20 (mitochondrial membrane protein marker) showed that si-RNA reduced the HIF-1α aggregation in mitochondria in hypoxia-stimulated chondrocytes (Fig. 3j). In summary, these significant data indicated that HIF-1α might play a protective role in chondrocytes survival in oxygen-deficient environment.

### DMOG increased HIF-1α expression, protects against hypoxia-induced apoptosis and autophagy flux

**Fig. 4:**
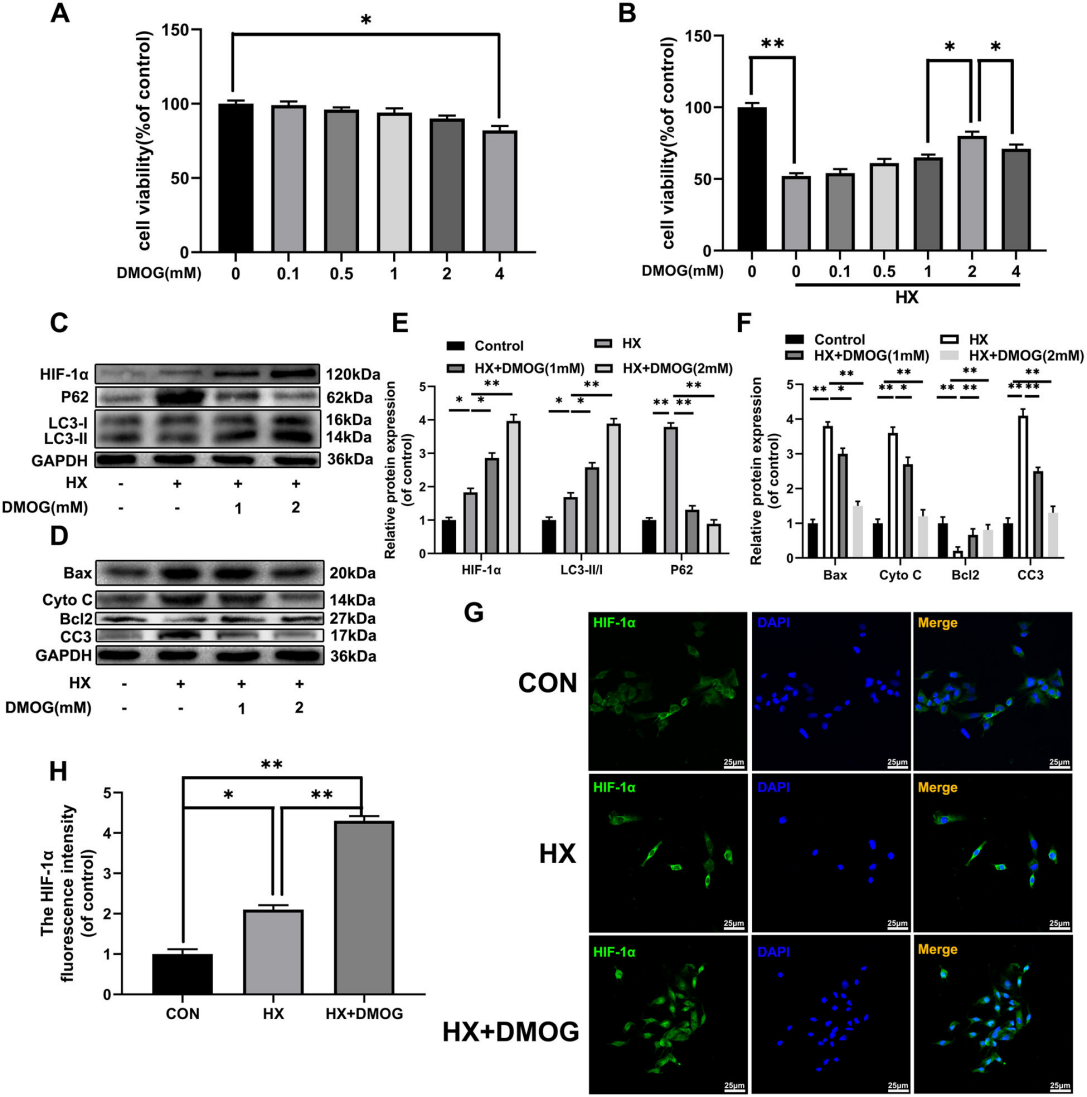
DMOG increases HIF-1α expression, protects against hypoxia-induced apoptosis and blocks autophagic flux. The chondrocytes were treated with DMOG and followed hypoxia treatment (1% pO_2_) for 24 h. **a**, **b** CCK-8 assay was used to determine the effect of increasing concentrations of DMOG alone or combined with hypoxia condition on the viability of chondrocyte cells. **c** The expression of HIF-1α, p62, and LC3 in chondrocytes. **d** The expression of Bax, Cyto C, Bcl2 and Cleaved-caspase3 in chondrocytes. **e**, **f** Quantification of immunoblots. **g** Image of immunofluorescence staining of HIF-1α; All data represent mean ± S.D. (*n* = 6). **P < 0.01, *P < 0.05.

Previous studies have shown that DMOG, a PHD inhibitor is a promising treatment for ischemic hypoxic disease through HIF-1α dependent mechanism. In our current investigation, we performed whether DMOG could increase chondrocyte survival and the therapeutic effects of HIF-1α in chondrocytes. Firstly, DMOG was not cytotoxic to chondrocytes at 0, 0.1, 0.5, 1, 2, 4 m concentrations, M after treatment for 24 h (Fig. 4a) by CCK-8. Furthermore, under hypoxic conditions, 1 mM and 2 mM DMOG concentrations maintained high cellular chondrocytes viability (Fig. 4b). Chondrocytes treated with the increasing DMOG concentration displayed enhancing HIF-1α level, suggesting that DMOG could increase the HIF-1α expression levels in chondrocytes. Similarly, DMOG activated autophagy by increasing LC3II, and the P62 which was significantly reduced under hypoxic condition, indicating that blocked autophagy flux was recovered. Also, the pro-apoptotic proteins (Cleaved-caspase3 and Bax) and mitochondria related protein cytochromes C (Cyto C) expression levels were significantly reduced with DMOG treatment while anti-apoptotic protein (Bcl-2) was markedly increased in DMOG group in a dose dependent manner (Fig. 4c–f). Fluorescence staining for HIF-1α in the chondrocytes showed that HIF-1α intensity was significantly increased after DMOG treatment (Fig. 4g, h). Thus, these significant results indicated that DMOG could increase HIF-1α expression, activate autophagy and suppress apoptosis in hypoxia stimulated chondrocytes.

### DMOG promoted mitophagy and inhibited mitochondrial dysfunction, apoptosis through regulating HIF-1α expression in hypoxia stimulated chondrocytes

**Fig. 5:**
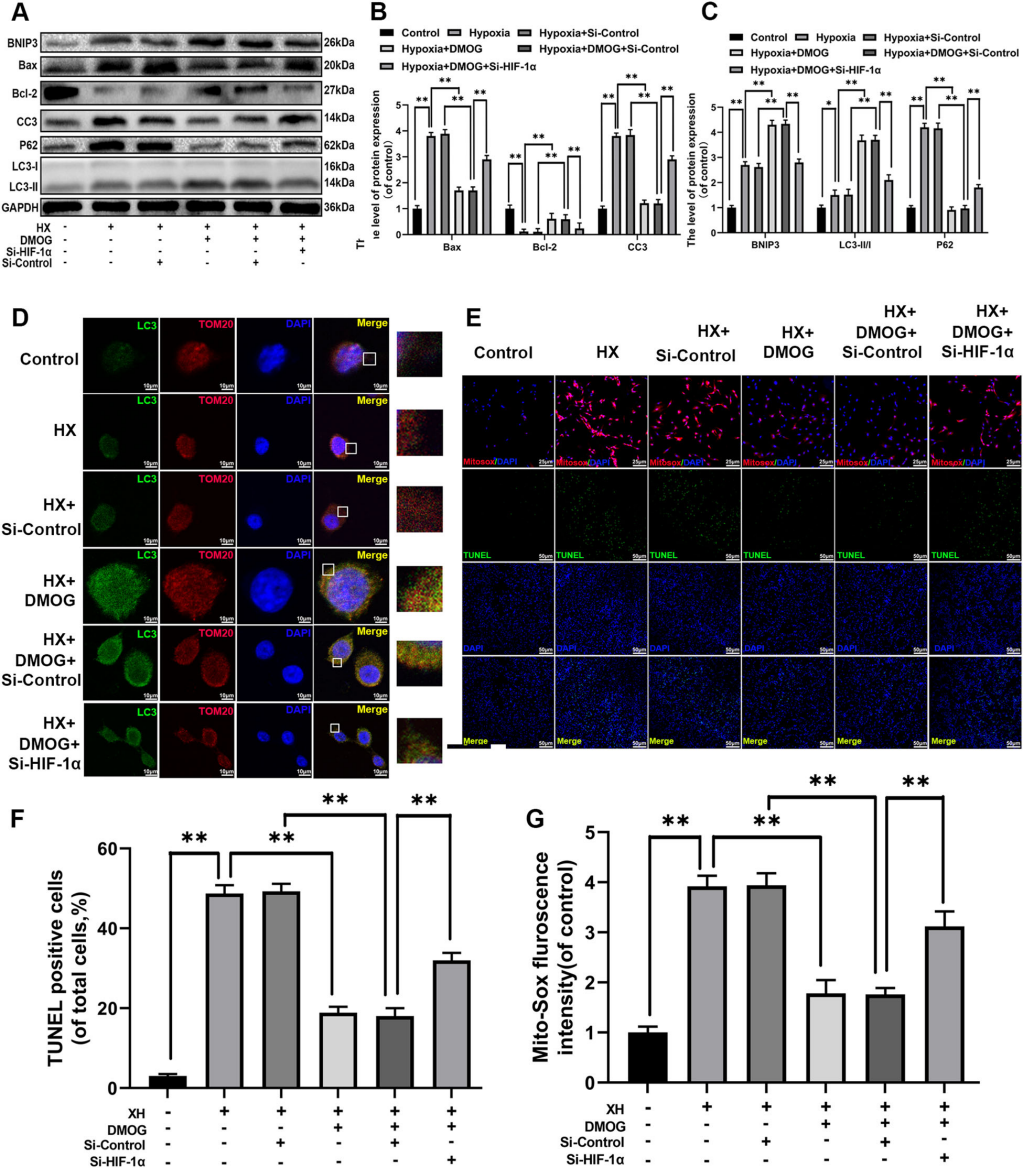
DMOG promotes mitophagy and inhibits mitochondrial dysfunction and apoptosis through regulating HIF-1α expression in hypoxia stimulated chondrocytes. The chondrocytes were pretreated with Si- HIF-1α for 48 h followed hypoxia treatment (1% pO_2_) and DMOG (2 mM) treatment for 24 h. **a** The expression of LC3-I, II, P62, BNIP3, Bcl-2, Bax, Cyto C and Cleaved-caspase3 from hypoxia treated chondrocytes were analyzed by western blot. **b**, **c** Quantification of LC3-I, II, P62, BNIP3, Cyto C, Bcl-2, Bax and Cleaved-caspase3 by immunoblots. **d** Representative image of immunofluorescence double staining of LC3 and Tom20 in chondrocytes. **e** Mito-Sox and TUNEL assay were performed in chondrocytes. **f** The fluorescence intensity of TUNEL in chondrocytes was quantified. **g** The fluorescence intensity of Mito-sox in chondrocytes was quantified. All data represent mean ± S.D. (*n* = 6). **P < 0.01, *P < 0.05.

In Fig. 4, the results showed that DMOG could markedly activate autophagy and inhibit apoptosis. However, the potential effect of DOMG on autophagy was depending on HIF-1α is unknown. Therefore, we knockdown the HIF-1α by siRNA to ascertain the mechanism of DMOG on mitophagy. After pretreated with HIF-1α siRNA, the ratio of LC3-II/LC3-I was sharply decreased, whereas the P62 expression was significantly increased in siRNA-HIF-1α group. The BNIP3 expression level was up-regulated by DOMG treatment, while HIF-1α knockdown effectively reduced the BNIP3 level relative to control (Fig. 5a, b). To determine whether DMOG could alleviate hypoxia induced mitochondrial dysfunction and apoptosis via increasing HIF-1α, we used western blot, TUNEL staining and Mito-Sox assay in vivo experiment. Bcl-2 expression levels were significantly decreased, while Bax and cleaved-caspase3 expression levels were remarkably increased in DMOG-treated chondrocytes with HIF-1α siRNA (Fig. 5a, c). Co-localization of autophagosomes could be used for assessing the level of mitophagy mediated clearance of depolarized/dysfunctional mitochondria in hypoxia stimulated chondrocytes. Autophagosomes and mitochondria were stained with LC3 and Tom20 (mitochondrial outer membrane marker), respectively. By double immunofluorescence staining with LC3-II and Tom20, we observed that DMOG discernibly increased the LC3 positive autophagosomes formation which were co-localized with the mitochondria compared to the hypoxia group, which was reversed by siRNA- HIF-1α (Fig. 5d). HIF-1α siRNA remarkably reversed the increase of Tunnel positive cells and fluorescence intensity of Mito-Sox (Fig. 5e–g). In summary, DMOG significantly alleviated the mitochondrial dysfunction and apoptosis under hypoxia stimulation through HIF-1α-mediated mitophagy.

### DMOG regulated ECM-associated and senescence-related proteins and genes via HIF-1α

**Fig. 6:**
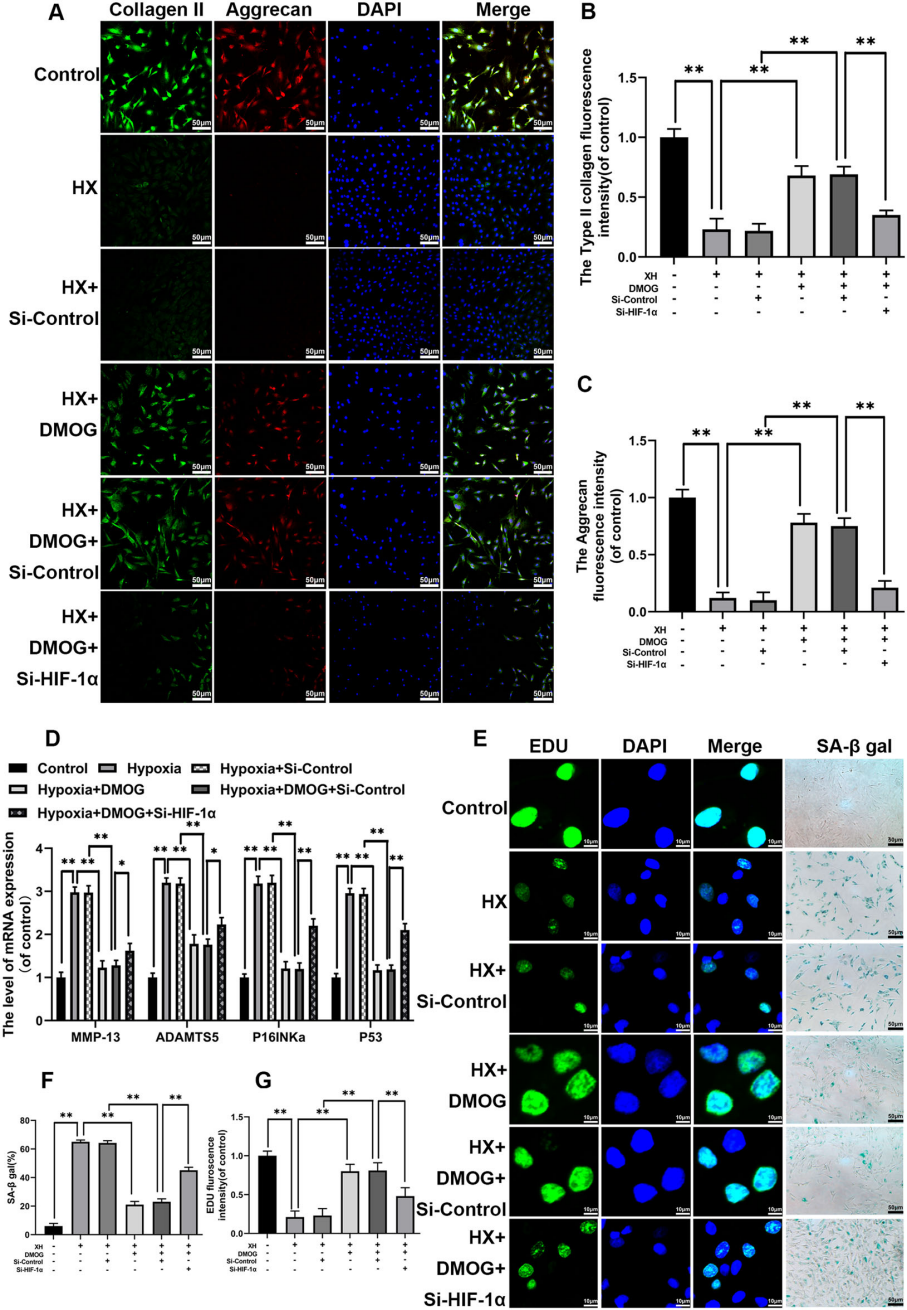
DMOG regulates the expression of ECM-associated and senescence-related proteins and genes via promoting HIF-1α expression. The chondrocytes were pretreated with Si-HIF-1α for 48 h followed hypoxia treatment (1% pO_2_) and DMOG (2 mM) treatment for 24 h. **a** The expression of Collagen II and Aggrecan were assessed by immunofluorescence. **b**, **c** The fluorescence intensity analyzed using Image J. **d** The mRNA expression of MMP-13, ADAMTS5, P16 and P53 in chondrocytes treated as above. **e**–**g** EDU assay and SA-β gal staining were performed in chondrocytes. All data represent mean ± S.D. (*n* = 6). **P < 0.01, *P < 0.05.

The P16, P53, MMP-13 and ADAMTS5 genes expression level were measured by RT-qPCR to explore whether HIF-1α participated in the cell senescence and ECM metabolism in chondrocytes. In addition, we detected the collagen II and aggrecan expressions by immunofluorescence. SA-β-galfor detecting senescent cells, and Edu assay for detecting the proliferation capacity were also assessed. As shown in Fig. 6d, the P16, P53, MMP-13 and ADAMTS5 protein expression levels were significantly enhanced in the hypoxia group compared to the other three groups. These genes were markedly reduced in chondrocytes with DMOG pretreatment for 2 h and another hypoxia induction for 24 h. HIF-α knockdown significantly reduced the potential effects of DMOG. Furthermore, hypoxia could decrease both aggrecan and collagen II expression levels, which were partly reversed by DMOG administration. HIF-1α-siRNA group also showed less aggrecan and collagen II fluorescence intensity (Fig. 6a–c). For cell senescence and proliferation capacity, DMOG could markedly rescue aging cell from senescence induced by hypoxia stimulation, and improve proliferation capacity. Furthermore, this potential effect was reversed by knockdown of HIF-1α (Fig. 6e–g). Thus, these important results indicated that DMOG could substantially inhibit the extracellular matrix degradation and cell aging through enhancing the HIF-1α.

### DMOG ameliorated OA development in DMM mouse model

**Fig. 7:**
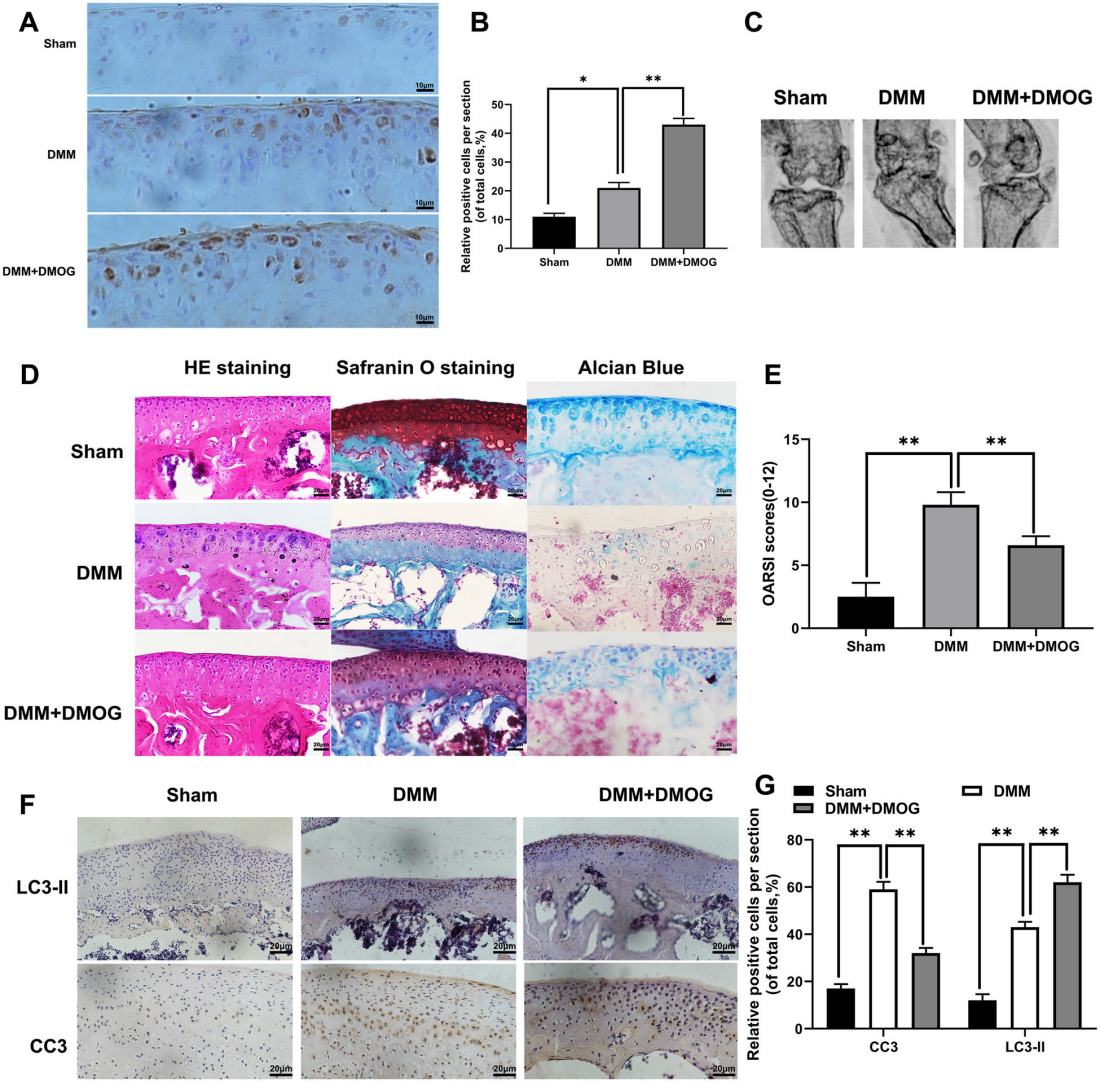
DMOG ameliorates OA development in DMM mouse model. Osteoarthritis mouse model was established by surgical destabilization of the medial meniscus and 8-week injuryknee jointwere takenX-ray, RT-qPCR and stained with H&E, Alcian Blue, Safranin Oandimmunohistochemistry staining. **a**, **b** The expression of HIF-1α was evaluated by immunohistochemistry staining. **c** Digital X-ray image of mouse knee joints in different groups. **d** Representative HE, S-O, Alcian Blue staining of cartilage in each group at 8-week after surgery. **e** OARIS scores of cartilage in each group. **f**, **g** The expression of LC3-II and Cleaved-caspase3 were evaluated by immunohistochemistry staining. All data represent mean ± S.D (*n* = 6). **P < 0.01, *P < 0.05.

According to the vitro experiments, we investigated the potential effects of DMOG on imageology and histomorphology of knee joints in DMM mouse model. HIF-1α immunohistochemical staining showed that DMOG could increase the HIF-1α expression levels in vivo model (Fig. 7a, b). X ray imaging obtained at 8-week after surgery showed that DMM group exhibited aberrantly joint space, however, lower narrow of joint space was found in DMM+DMOG group (Fig. 7c). Erosion and hypo-cellularity of the superficial articular cartilage, and proteoglycan loss were observed in DMM group by Safranin O staining, HE staining and Alcian Blue staining at 8-week after surgery. In contrast, the DMM+DOMG group had more complete cartilage surface, and richer proteoglycan (Fig. 7d). Consistent with staining, OARSI score was declined by DMOG, and rising in DMM group (Fig. 7e). Meanwhile, in order to determine the effect of HIF-1α-mediated mitophagy on apoptosis in vivo, we detected the LC3II, and Cleaved-caspase3 expression levels by immunohistochemical staining. The results showed that increased DMOG induced HIF-1α promoted LC3 expression and decreased Cleaved-caspase3 expression in chondrocytes (Fig. 7f, g). Therefore, these important findings revealed that DMOG-induced up-expression of HIF-1α could alleviate osteoarthritis via activation of autophagy.

## Discussion

OA is one of most prevalent musculoskeletal diseases and it is regarded as a central cause disability worldwide^31,32^. OA leads to the articular cartilage degeneration and variation in subchondral bone^33^. In addition, it is accompanied by serious synovium inflammation and irreversible cartilage degradation, with the following major characteristics: progressive differentiation and maturity of the articular cartilage, progressive degradation of the extracellular matrix, progressive loss of the articular cartilage components^34^. From the perspective of cell morphology, a large number of apoptotic and senescent chondrocytes are the main cytological features of OA^35,36^. Extensive studies have documented that mitophagy leads to several diseases progression, including cancer^37^, neurodegenerative diseases^38^ and cardiovascular disease^39^. Here, we sought to establish the potential role of HIF-1α-mediated mitophagy in eliminating dysfunctional mitochondria and its impact on remitting apoptosis and senescence in chondrocyte induced by hypoxia condition.

HIF-1α is a transcription factor acting as a physiological regulator, which maintains homeostasis in chondrocytes as well other cell types^40,41^. In normoxic conditions, prolyl hydroxylases (PHDs) hydroxylates HIF-1α on proline residues (Pro402/564) in the oxygen-dependent degradation (ODD) domain. This hydroxylation process allows the HIF-1α recognition by the von Hippel-Lindau (pVHL), a kind of tumor-suppressor protein and the substrate recognition component of an E3 ubiquitin ligase complex that aims HIF-α for proteasomal degradation^42,43^. On the contrary, under hypoxic conditions, HIF-1α hydroxylation is suppressed accumulating in the cytoplasm, allowing its phosphorylation and subsequent translocation to the nucleus, contributing to transcribing downstream target genes.

In current study, we found that HIF-1α expression was notably increased in the OA human articular cartilage, mouse knee articular cartilage of DMM model and hypoxia treated rat chondrocytes. The dysregulated mRNAs identified in hypoxia treated rat chondrocytes by transcriptional analysis were enriched in several signaling pathways, including metabolic pathways, autophagy, apoptosis, senescence and the HIF-1α signaling pathways. Furthermore, core mRNA BNIP3 was significantly enriched, a major target of HIF-1α transcriptional genes and regarded as important mitophagy receptors and adaptors. Therefore, we hypothesized that HIF-1α could alleviate hypoxia-induced apoptosis, senescence and matrix degradation in chondrocytes through mitophagy via enhancing BNIP3 expression. Although the protective effects of HIF-1α in osteoarthritis and rheumatoid arthritis in mice have been documented, there is no any clear data in this field showing the differential expression of HIF-1α in the normal people and patients with osteoarthritis. As mentioned earlier, hypoxia leads to change in mitochondria, including decreased oxidative phosphorylation level, cytochrome c oxidase activity and increased ROS production. Among them, intracellular ROS are derived from mitochondria and membrane NADPH oxidase (Nox) system. Over-induced ROS is tightly related to age-related diseases, including OA and IDD^44–46^.

In our current study, the HIF-1α expression was markedly elevated in the hypoxia-induced mice chondrocytes, together with apoptosis, cell senescence, ECM metabolic unbalance, and increased ROS and autophagy. Subsequent experiments showed that HIF-1α regulated the eliminating hypoxia-induced cell damage and played an important role in ROS production. Interestingly, DMOG could increase HIF-1α expression in both vitro and vivo, showing significant therapeutic effects against apoptosis and senescence. PHD is an important modulator of HIF-1α, could stabilize the HIF-1α during hypoxia, indicating that PHD inhibitors may become potential candidates for intervening senescence and apoptosis^47^ To investigate the protective effect of HIF-1α and the mechanism of its agonist DMOG, we performed the DMOG and HIF-1α siRNA to regulate the HIF-1α expression in chondrocytes. Our results suggested that the increased HIF-1α expression could improve the chondrocytes survival^48,49^.

Autophagy has been regarded as a protective mechanism under several pathological conditions through degrading useless proteins and organelles or dysfunctional organelles to maintain cellular functions^50^. In our current study, autophagy played an important role in preventing apoptosis and aging of chondrocytes to postpone OA. As a transcription factor and an oxygen sensor, phosphorylated HIF-1α could hypoxia-mediated sensitive genes in nucleus. Among them, SOX9 and COL2A1 regulate chondrocyte function and maintain ECM anabolism. HIF-1 could elevate type II collagen and Sox9 activity via activating hypoxia-induced transcriptional targeted genes^51,52^. The stabilized HIF-1α also could enhance the native type II collagen accumulation in the primary human chondrocytes, which could be inhibited by 2ME2^53^. Therefore, these substantial findings suggested that HIF-1α played an important role in maintaining the extracellular matrix integrity in articular cartilage. Furthermore, we found that HIF-1α not only enhanced the extracellular matrix synthesis at the transcriptional level, but also inhibited the hypoxia-induced extracellular matrix degradation through enhancing mitophagy.

To explore the mechanism of HIF-1α on mitophagy activation, we investigated the expression HiF-1α/BNIP3 classical signaling pathway in the chondrocytes treated with DMOG under hypoxia. The BNIP3 expression is necessary for hypoxia-induced autophagy activation and BNIP3 knockdown aggravated cell death during hypoxia^54,55^. In agreement with these previous studies, our study showed that the BNIP3 expression increased in a dose-dependent manner following the DMOG-induced HIF-1α expression. The siRNA HIF-1α could significantly reduce the BNIP3 expression and mitophagy, indicating that HIF-1α/BNIP3 was a significant mechanism involved in DMOG protective effect.

HIF-1α agonists, such as small molecule hypoxia-inducing factor-prolyl hydroxylase inhibitor GSK1278863, used in clinical trials to treat anemia caused by chronic kidney disease without serious adverse effects^50^. In addition, HIF-1α has shown potential therapeutic effects in cancer and fracture repair. Therefore, HIF-1α might be a potential target for alleviating OA.

We demonstrated that HIF-1α-mediated autophagy played a protective role in hypoxia-stimulated chondrocytes. However, precise mechanism on HIF-1α inhibited apoptosis and senescence was unknown. The protective effect of HIF-1α through inducing the transcription of anti-senescence and anti-apoptosis genes remained unclear. In the hypoxic environment, autophagy mechanism of reducing senescence and apoptosis needs to be further studied.

In conclusion, HIF-1α was related to the OA progression, regulated mitochondrial dysfunctions via mitophagy in the damaged chondrocytes. DMOG could significantly inhibit apoptosis and chondrocytes senescence, and recover ECM metabolic unbalance in the chondrocytes via HIF-1α mediated mitophagy.

## Materials and methods

### Ethics statement

All surgical interventions, treatments and postoperative animal care procedures were strictly performed accordance with the Animal Care and Use Committee of Wenzhou Medical University (ethic code: wydw2014-0129). The Human articular cartilage tissue collection and the experiments were approved by Ethical Committee of the Second Affiliated Hospital, Wenzhou Medical University and following the guidelines of the Declaration of Helsinki^56^.

### Human cartilage and chondrocytes culture

The normal human articular cartilages from 6 donors with no clinical symptoms and imaging features of OA were obtained from femoral condyles and tibial plateaus at autopsy (45–64 years old; mean, 53.8 years; Kellgren–Lawrence grade, 0 or I; *n* = 6). The OA human articular cartilages were achieved from 6 patients (45–60 years old; mean, 56.1 years; Kellgren–Lawrence grade, III or IV; *n* = 6) undergoing total knee arthroplasty. For the proposed activities, enrollment of specimen donors was performed by a strict procedure that has been approved by the Ethical Committee of the Second Affiliated Hospital, Wenzhou Medical University at participating institutions, with informed consent and the utmost attention to the issues of patient safety, anonymity and confidentiality. Human articular cartilages samples were cut into 1cm3 pieces and incubated with 2 mg/ml of collagenase II in DMEM at 37 °C for 4 h. After washing by PBS for 3 times and resuspension, the chondrocytes from cartilage were cultured in cell culture dish at a seeding density of 3 × 10^5^ cells per ml in DMEM/F12 contained with 10% FBS and 1% antibiotic in 5% CO_2_ at 37 °C. Cells from the second passage were used in subsequent experiments.

### Primary chondrocytes culture

Twelve healthy C57BL/6 mice (6 males and 6 females, 5 days) were euthanized with sodium pentobarbital overdose. The cartilage of the knee joint of mice was carefully removed under microscope in a sterile environment. Then, the tissues were digested with 2 mg/ml (0.1%) collagenase II for 4 h at 37 °C. Next, the digested tissue pieces were suspended and seeded into tissue culture flasks. The chondrocytes planted and expended in the condition of DMEM/F12 (Gibco, Invitrogen, Grand Island, NY) with 10% fetal bovine serum (FBS; Hyclone, Thermo Scientifc, Logan, UT, USA) and 1% penicillin/streptomycin antibiotics (Gibco, Invitrogen, Grand Island, NY) in the incubator with 5% CO_2_ at 37 °C. The medium was changed 3 times per week. Cultures were observed daily with an inverted phase contrast microscope. Cells from the second passage were suited for next experiments. All cells were tested negative for mycoplasma contamination.

### Hypoxic cell model

Cells in good condition at logarithmic growth stage were selected, digested with 0.25% trypsin, centrifuged, supernatant removed, re-suspended and inoculated in cell culture plates for the corresponding experiment. The normal oxygen group was cultured in the cell incubator. The hypoxic group and the drug administration group were placed in the modular incubator chamber, and were given secondary vitality containing different concentrations of N_2_ and CO_2_. Experiments were carried out when the oxygen concentration in the incubator was maintained to the target concentration.

### RNA-sequencing and bioinformatics analysis

Primary chondrocytes (1 × 10^5^ cells per well) were seeded in 6-well plates, grown to 80% confluence and treated with hypoxia condition (1% pO_2_) for 1 day. Then, total RNA from cells of each group was extracted using TRIzol reagent (Invitrogen) according to the manufacturer’s instructions. RNA-sequencing and bioinformatics analyses were performed as previously described^57^.

### Si-RNA transfections

Effective siRNAs for HIF-1α and control siRNA were obtained from Invitrogen (Carlsbad, CA, USA). When reaching 50–60% confluence cells, chondrocytes were transfected with HIF-1α siRNA using Lipofectamine 2000 siRNA transfection reagent (Thermo Fisher, UT, USA) according to the manufacturer’s protocols.

### Cell viability assay

Cell viability was detected using CCK8 according to the manufacturer’s instructions. Briefly, chondrocytes were planted into the 96 well-plate (8000 cells/well) and incubated in DMEM/F12 with 10% FBS at 37 °C for 24 h. Then, chondrocytes were treated with the different concentration of DMOG as described above. A total of 10 µL CCK-8 solution was added into each well. And the plate was incubated for an additional 1 h. The absorbance of samples was measured at 450 nm using a microplate reader (Thermo Scientific, USA).

### Western blot analysis

After isolating total protein from chondrocytes using RIPA buffer with 1 mM phenylmethanesulfonylfluoride (PMSF), the protein concentration was measured by BCA protein assay kit (Beyotime). Protein samples were separated by sodium dodecyl sulfatepolyacrylamide gel electrophoresis (SDS-PAGE) and then transferred to a 0.45μm polyvinylidene difluoride membranes (Millipore, USA). Following blocking with 5% non-fat milk for 2 h, the membranes were incubated with primary antibodies against HIF-1α (1:500), cleaved-caspase3 (1:1000), Bax (1:1000), Bcl-2 (1:1000), Cyto C (1:1000), LC3 (1:1000), p62 (1:1000), GAPDH (1:1000), and BNIP3 (1:500) overnight at 4 °C. Then, bands were incubated with the respective secondary antibodies. Finally, the bands were detected using electrochemiluminescence plus reagent (Invitrogen). Image Lab 3.0 software (Bio-Rad) was used to quantified the intensity of protein bands.

### Real-time PCR

The total RNA was extracted using TRIzol reagent (Invitrogen, Grand Island, NY). One microgram of total RNA was used to synthesize cDNA (MBI Fermantas, Germany). For quantitative real-time PCR (qPCR), a total 10 μl of reaction volume, including 5 μl of 2× SYBR Master Mix, 0.25 μl of each primer and 4.5 μl of diluted cDNA was used. The machine parameters of RT-PCR were: 10 min 95 °C followed by 40 cycles of 15 s 95 °C and 1 min 60 °C. The reaction was performed using CFX96Real-Time PCR System (Bio-Rad Laboratories, California, USA). The cycle threshold (Ct) values were collected and normalized to the GAPDH level. The results were assessed by using the 2−ΔΔCt method. The primer sequences were as follow: for MMP-13, Forward 5′-GTGATGATGATGATGATGAC-3′, Reverse 5′-GCAGGA TGG TAGTATGATT-3′; for ADAMTS5, Forward 5′-TCTCCAAAGGTTACGGATGGGR-3′, Reverse 5′-TCTTCTTCAGGGCTAAGTAGGCAG-3′; for P16, Forward 5′-CTCCTGAAAATCAAGGGTTGAG-3′, Reverse 3′-ACCTTCCTAACTGCCAAATTGA-5′; for P53, Forward 5′-GTGAGGGATGTTTGGGAGATG-3′, Reverse 5′- CCTGGTTAGTACGGTGAAGTG-3′.

### Immunohistochemistry

After double deparaffinization and hydration through xylene and graded alcohol series, sample sections were incubated with 3% H_2_O_2_ for 10 min and washed by PBS for 3 times. Then, the tissue sections were incubated with 0.1% trypsin for 20 min and washed by PBS for 3 times. After blocking with 10% (w/v) BSA for 1 h at 37 °C, the sections were incubated with specific primary HIF-1α, LC3II, Cleaved-caspase3 antibody (Abcam, USA, 1:200) at 4 °C overnight. Negative control sections were incubated with non-specific IgG. After incubation at 37 °C for 1 h, the tissue sections were washed by PBS for 3 times and incubated with HRP-conjugated secondary antibodies for 1 h at 37 °C. Color development was performed using a DAB color development kit (ZhongShan Biotech). At least 3 sections from each specimen were observed. The rate of positive cells each section was measured by observers who were blinded to the experimental groups. The positive points were quantified by Image J software 2.1 (Bethesda, MD, USA).

### Immunofluorescence

Cells were fixed on coverslips, and blocked by 10% goat serum for 1 h at 37 °C. Samples were then probed using primary antibodies HIF-1α (1:100, Abcam), Tom20 (1:100, Santa Cruz Biotechnology), rabbit anti-LC3 (1:100, Cell Signaling Technology), Collagen type II (1:100, Abcam) and Aggrecan (Santa Cruz Biotechnology, 1:50) at 4 °C overnight. Then slides were incubated with fluorescein isothiocyanate or tetramethyl rhodamine isothiocyanate conjugated second antibodies for 45 min and labeled with 40, 6-diamidino-2-phenylindole (DAPI, Beyotime) for 3 min. finally, slides were assessed in a confocal fluorescence microscope (Nikon, Japan). In addition, fluorescence intensity was measured using Image J software 2.1 (Bethesda, MD, USA).

### Measurement of mitochondrial Reactive oxygen species (ROS)

Mitochondrial ROS was measured by using Mito-SOX Red dye (Invitrogen, M36008). NP cells were plated in six-well plates. After treated above, cells were incubated with Mito-Sox Red (5 μM) for 30 min at 37 °C, then washed by PBS for 2 times, and observed by the confocal fluorescence microscope (Nikon, Japan).

### Mitochondrial membrane potential (ΔѰM)

ΔѰM was assessed using the mitochondrial-specific fluorescent probe JC-1 (5, 5′, 6, 6′-124 tetrachloro-1, 1′, 3, 3′- tetraethyl-benzimidazolylcarbocyanine iodide) (Yeasen Biochemical, Shanghai, China). Brief, cartilage cells were incubated with JC-1 (5 μM) for 30 min at 37 °C and washed by PBS for 3 times and observed by the Flow cytometer.

### Mito-tracker red staining

Mito-tracker red CMXRos (Yeasen Biochemical, Shanghai, China) was used to determine the mitochondria level in live cells. According to the manufacturer’s instructions, cartilage cells were incubated with Mito-tracker probes at the 50 nM concentration for 30 min at 37 °C. Then, cell nuclei were stained with DAPI for 5 min at 37 °C. NP cells were washed by PBS for 3 times and imaged with the confocal fluorescence microscope (Nikon, Japan). The fluorescence intensity was quantified by using Image J software 2.1 (Bethesda, MD, USA).

### SA-β-gal staining

Senescence level was measured by senescence associated β-galactosidase (SA-β-gal) staining kit (Beyotime, Shanghai, China) according to the manufacturer’s instructions. Aging chondrocytes showing higher SA-β-gal activity were stained blue.

### TUNEL staining

TUNEL staining was used to detect the damaged DNA level. Chondrocytes were fixed and then stained with in situ cell death detection kit (Yeasen Biochemical, Shanghai, China) according to the manufacturer’s instructions for 30 min at 37 °C and the nuclei was stained with DAPI. Twelve fields of each slide were randomly selected and captured under Nikon ECLIPSE Ti microscope to count TUNEL positive cells.

### Animal model

Sixty 10-week-old C57BL/6 mice (30 male and 30 female) were purchased from Animal Center of Chinese Academy of Sciences (Shanghai, China) and were divided randomly into the sham group, DMM group and DMM+DMOG group. OA mouse model was established by surgical destabilization of the medial meniscus as previously described^58^. For DMM+DMOG group, DMOG was administrated to animals by intraperitoneal (i.p.) injection at 25 mg/kg per day. Mice were sacrificed at 8 weeks post-OA surgery from each group. The knee joints were dissected and processed for histological evaluation. The investigator was blinded to the group allocation during the experiment and when assessing the outcome.

### X-ray imaging method

After 8 weeks of surgery, the animals were given the X-ray examination. X-ray imaging was performed on all mice to evaluate the joint space using a digital X-ray machine (Kubtec Model XPERT.8; KUB Technologies). Proper images were obtained in the following settings: 50 Kv and 160 µA.

### Histopathological analysis

The mice were sacrificed by 10% chloral hydrate intraperitoneal injection and the knee joint were harvested 8 weeks after surgery. The specimens were fixed in 4% (v/v) paraformaldehyde for 24 h and decalcified in 10% (v/v) ETDA. After dehydrated, tissues were embedded in paraffin and cut into 5-μm sections. The sample slides were stained with HE staining, safranin O-fast green (S-O) and Alcian Blue. Mice in each group were evaluated by Osteoarthritis Research Society International (OARSI) scoring system for medial femoral condyle and medial tibial plateau.

### Statistical analysis

The results were expressed as mean ± S.D. Raw statistical analyses were processed by using Graphpad Prism 6 (USA). Nonparametric data were analyzed by Mann–Whitney *U*-test. Data were analyzed by one-way analysis of variance (ANOVA) followed by the Tukey’s test for comparison between different groups. All the experiments were performed in six times and were consistently repeatable.

## Supplementary information

Fig. S1

Supplemental Materials and Methods

Supplementary Figure Legends
