# Supplementary figures and images for: Stabilization of HIF-1α alleviates osteoarthritis via enhancing mitophagy
Source: Cell Death Dis. 2020 Jun 25;11(6):481. doi: 10.1038/s41419-020-2680-0 (PMC7316774; doi:10.1038/s41419-020-2680-0)

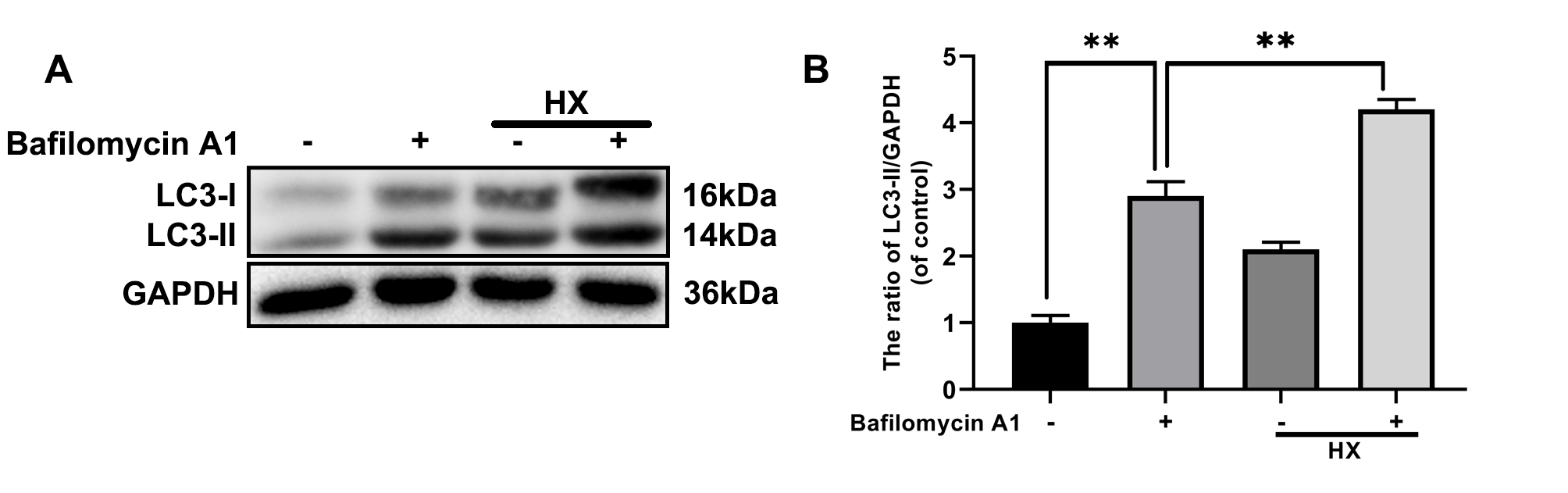

Supplement: Supplementary file 1 — Fig. S1 [file 41419_2020_2680_MOESM1_ESM.tif]
