# Supplementary material for: Stabilization of HIF-1α alleviates osteoarthritis via enhancing mitophagy
Source: Cell Death Dis. 2020 Jun 25;11(6):481. doi: 10.1038/s41419-020-2680-0 (PMC7316774; doi:10.1038/s41419-020-2680-0)
Supplement: Supplementary file 2 — Supplemental Materials and Methods [file 41419_2020_2680_MOESM2_ESM.docx]

**Materials and Methods**

**RNA library construction and bioinformatics**

The extracted RNA was subjected to quality control assessments and the mRNA was enriched with mRNA capture beads. Then first-strand cDNA was synthesized using the mRNA as template and random hexamers as primers, the second strand of cDNA was subsequently generated using RNase H and DNA polymerase I. The double strand cDNA was purified by VAHTSTM DNA clean beads. The purified cDNA was repaired at both termini, added with poly-A tails and sequencing adaptors. The resulting cDNA fragments were then size-selected using VAHTSTM DNA Clean Beads and amplified by PCR. The purified PCR products constituted the libraries to be sequenced, which underwent another round of quality control examination on an Agilent 2100 Bioanalyzer. Finally, the libraries were sequenced using the Illumina HiSeq platform^1^.

The original sequencing data (raw reads) were prefiltered to remove the reads with adapters, the reads containing more than 5% unidentifiable bases, and the reads comprising more than 50% bases with the mass value less than 10 (Q ≤ 10), whereby the high-quality sequencing data (clean reads) were obtained. Then the clean reads with a minimum 200-bp overlaps were aligned to the reference sequences, which subserved the second quality control of bioinformatics analysis. The results of this quality control depended upon the distribution and coverage of the clean reads on the reference sequences.

Subsequently, the analyses of differentially expressed genes (DEGs) was performed. The abundance of each gene transcript was measured by the number of fragments per kilo bases per million reads, which was calculated by the software Cufflink v2.1.1. In addition, the GO enrichment and KEGG pathways were analysed.

**References**

1 Y, L. *et al.* RNA-Seq identifies novel myocardial gene expression signatures of heart failure. *Genomics* **105**, 83-89, doi:10.1016/j.ygeno.2014.12.002 (2015).
