# Supplementary material for: Stabilization of HIF-1α alleviates osteoarthritis via enhancing mitophagy
Source: Cell Death Dis. 2020 Jun 25;11(6):481. doi: 10.1038/s41419-020-2680-0 (PMC7316774; doi:10.1038/s41419-020-2680-0)
Supplement: Supplementary file 3 — Supplementary Figure Legends [file 41419_2020_2680_MOESM3_ESM.docx]

**Supplementary Figure Legend**

**Fig. S1 Effects of hypoxia condition-induced autophagic flux dysfunction in chondrocytes** The chondrocytes were pretreated with bafilomycin followed hypoxia treatment (1% pO_2_) for 24 h **a** The expression of LC3-II, II was evaluated by western blots. **b** Quantification of LC3-II, II immunoblots.
